# Molecular variation among virulent and avirulent strains of the quarantine nematode *Bursaphelenchus xylophilus*

**DOI:** 10.1007/s00438-020-01739-w

**Published:** 2020-11-10

**Authors:** Anna Filipiak, Tadeusz Malewski, Ewa Matczyńska, Marek Tomalak

**Affiliations:** 1 Department of Biological Pest Control, Institute of Plant Protection, National Research Institute, Władysława Węgorka 20, 60-318 Poznan, Poland; 2 Department of Molecular and Biometric Techniques, Museum and Institute of Zoology, Polish Academy of Sciences, Wilcza 64, 00-679 Warsaw, Poland; 3 Genomed SA, Ponczowa 12, 02-971 Warsaw, Poland

**Keywords:** Pine wood nematode, Pine wilt disease, Next-generation sequencing, Genome variation, GO-enrichment analysis

## Abstract

*Bursaphelenchus xylophilus* is an emerging pathogenic nematode that is responsible for a devastating epidemic of pine wilt disease worldwide, causing severe ecological damage and economic losses to forestry. Two forms of this nematode have been reported, i.e., with strong and weak virulence, commonly referred as virulent and avirulent strains. However, the pathogenicity-related genes of *B. xylophilus* are not sufficiently characterized. In this study, to find pathogenesis related genes we re-sequenced and compared genomes of two virulent and two avirulent populations. We identified genes affected by genomic variation, and functional annotation of those genes indicated that some of them might play potential roles in pathogenesis. The performed analysis showed that both avirulent populations differed from the virulent ones by 1576 genes with high impact variants. Demonstration of genetic differences between virulent and avirulent strains will provide effective methods to distinguish these two nematode virulence forms at the molecular level. The reported results provide basic information that can facilitate development of a better diagnosis for *B. xylophilus* isolates/strains which present different levels of virulence and better understanding of the molecular mechanism involved in the development of the PWD.

**Electronic supplementary material:**

The online version of this article (10.1007/s00438-020-01739-w) contains supplementary material, which is available to authorized users.

## Introduction

Pine wilt disease (PWD) is one of the most serious global tree diseases affecting coniferous forests around the world. It is caused by the pine wood nematode (PWN), *Bursaphelenchus xylophilus* (Nematoda: Aphelenchoididae) (Mota and Vieira 2008; Jones et al. 2013). This nematode is listed as a major plant quarantine organism for most countries in the world (Evans et al. 1996; Futai 2013). *B. xylophilus* is native to North America where it causes only a limited damage to native pines, although non-native species are profoundly affected (Jones et al. 2013). PWN was introduced into Japan during the early twentieth century and it spread subsequently to other East Asian (China, Taiwan, South Korea) and European (Portugal, Spain) countries (Mota et al. 1999; Robertson et al. 2011; Futai 2013; Ding et al. 2016; Filipiak et al. 2017). The nematodes are transmitted to healthy trees by longhorn beetles in the genus *Monochamus*, during maturation feeding of the insect (Togashi and Shigesada 2006). Once *B. xylophilus* enters the pine tree, it migrates through the resin canals, destructively feeding on the parenchymal cells (Shinya et al. 2013). In many regions worldwide pine is among the most important tree genera for the forest industry and the rapid spread of this disease has become a major problem (Mota and Vieira 2008; Vicente et al. 2012; Espada et al. 2016). Every year thousands of trees displaying symptoms of PWD are felled and removed in the affected areas. *B. xylophilus* causes the death of host trees in less than one year after infection under suitable environmental conditions, and the risk of this problem is likely to increase due to climate changes (Pereira et al. 2013; Qiu et al. 2013).

Earlier research provided evidence that *B. xylophilus* could effectively compete for the host tree and vector insects with native species *B. mucronatus*, and reduce or displace it from the original locations (Futai 1980; Cheng et al. 2009). However, in our in vitro and in vivo research conducted in plate cultures of *Botryotinia fuckeliana* and pine seedlings, respectively, a range of variation in results of such competition was observed among particular isolates of these two competing nematodes (Tomalak and Filipiak 2013).

Kiyohara and Bolla (1990) reported a great variation in the virulence level of *B. xylophilus* collected throughout Japan. Mortality of pine seedlings ranged from 0 to 100% and the virulence of nematode isolates from a single stand of pines also varied significantly. With respect to pathogenicity, two major groups of strains, i.e., virulent and avirulent, have been reported in *B. xylophilus* (Bolla and Boschert 1993; Akiba et al. 2012). To date, many studies have reported that the virulence level of *B. xylophilus* is associated with its reproductive potential and population growth ability (Kiyohara and Bolla 1990; Aikawa et al. 2003; Wang et al. 2005; Aikawa and Kikuchi 2007; Shinya et al. 2012; Qiu et al. 2013; Filipiak 2015). The generation time of virulent isolates cultured virulent on *B. fuckeliana* at 25 °C was shorter than that of avirulent isolates and the rate of population increase was faster than in the avirulent isolates (Wang et al. 2005). In the in vivo situation, when virulent and avirulent isolates were separately inoculated into *Pinus thunbergii* seedlings, nematode density of the virulent population increased with time after inoculation, while the avirulent isolate never reproduced (Kiyohara and Bolla 1990; Aikawa and Kikuchi 2007). Thus, the results of the earlier studies suggest that virulence is closely associated with the nematode reproductive potential, irrespective of in vitro or in vivo conditions.

In PWD the mechanism of pathogenicity is complicated and involves many pathogenic factors including host pines, nematodes, beetles, fungi, bacteria, environmental factors, and other aspects (Shinya et al. 2013). A draft genome sequence of *B. xylophilus* was reported in 2011 (Kikuchi et al. 2011). Its availability should facilitate additional studies on *B. xylophilus* pathogenicity. To date, however, only a few transcriptome-type studies (Espada et al. 2016; Li et al. 2019, 2020; Hu et al. 2020) and only one genome-wide analysis (Palomares-Rius et al. 2015) have been reported.

The main objective of this study was to reveal genetic differences related to pathogenicity of *B. xylophilus*. It was achieved by resequencing of four *B. xylophilus* strains followed by comparison and analysis of the complete nuclear genome sequences of two virulent (BxPt67OL and BxMad24C) and two avirulent (C14-5 and OKD-1) populations of this nematode.

## Materials and methods

### Nematode strains

In the reported study, two virulent and two avirulent strains of *B. xylophilus* were examined. The origin and ecological features of the nematode strains are summarized in Table 1.

**Table 1:** Origin of *Bursaphelenchus xylophilus* strains used in the study, and their virulence

Strain name	Geographical origin	Virulence
BxPt67OL	Portugal	Virulent
BxMad24C	Madeira, Portugal	Virulent
C14-5	Chiba, Japan	Avirulent
OKD-1	Okayama, Japan	Avirulent

The avirulent “C14-5” and “OKD-1” strains were kindly supplied by Dr. Y. Takeuchi from Kyoto University, Japan. The virulent “BxMad24C” and “BxPt67OL” strains were provided by Prof. M. Mota from the University of Évora, Portugal.

The virulence of the strains was demonstrated by previous inoculation tests on pine seedlings (Aikawa et al. 2003; Filipiak 2015). Prior to the examination, all strains were cultured on *B. fuckeliana*/potato dextrose agar at 25 °C for ca. 2 weeks. Propagated nematodes were stored in 20 μl distilled H_2_O at − 20 °C until being used for subsequent DNA extraction.

### Genomic DNA preparation and sequencing

Genomic DNA of nematodes was isolated with a QIAamp DNA Micro Kit (Qiagen, Hilden, Germany) according to the protocol provided by the manufacturer. DNA concentration and its purity were measured using a NanoDrop spectrophotometer (Thermo Fisher Scientific Inc., MA, USA). 50–100 ng of DNA was used to construct 350-bp libraries, using a TruSeq Nano DNA Library Prep Kit (Illumina, San Diego, USA) with the standard protocol. Libraries were sequenced on an Illumina HiSeqX Ten, according to the manufacturer’s recommended protocol, to produce 150-bp paired end reads (Genomed, Warsaw, Poland).

### Data analysis

The quality of the obtained raw sequencing data was first assessed by FastQC (Andrews 2010; https://www.bioinformatics.babraham.ac.uk/projects/fastqc). Subsequently, adapters and low quality bases were removed by Trimmomatic in paired-end mode (Bolger et al. 2014). For downstream analyses, only reads of a minimum length of 30 bp and Phred score > 30 were used.

#### Mapping

For each strain, Illumina reads were mapped to the *B. xylophilus* reference Ka4C1 strain genome (GCA_000231135.1) using Burrows–Wheeler Aligner (Li and Durbin 2009). Single nucleotide variants (SNVs) and small indels were determined using GATK HaplotypeCaller (Van der Auwera et al. 2013; https://gatk.broadinstitute.org/hc/en-us). Only variants with minimal Phred quality 30 and minimal coverage of 4 × where subjected for further analysis.

#### Variant effect analysis

Variant consequences for genes where evaluated with the ensembl variant effect predictor (VEP) (McLaren et al. 2016). Then, genes with one high impact variant which were present in at least one virulent populations, but absent in any of the avirulent populations were selected. Similarly, genes present in at least one avirulent population but absent in virulent ones were selected for further analysis. The calculations for each set of genes were made using in-house Linux tools and Python scripts. This approach allowed for identification of gene sets, which were further examined with Gene Ontology enrichment analysis.

#### GO-enrichment analysis

The WormBase database was used to determine the nematode gene annotations related to their functions (Gene Ontology Consortium 2004; https://geneontology.org/docs/ontology-documentation). Subsequently, using the topGO package from the R environment (https://bioconductor.org/packages/release/bioc/html/topGO.html), GO-enrichment analyzes were performed for both of the above gene sets. The topGO package is designed to facilitate semi-automated enrichment analysis for Gene Ontology (GO) terms. Each GO category (molecular function—MF, cellular component—CC, biological process—BP) is tested independently. GO enrichment analysis allows testing the over-representation of GO terms. In our study, we used two methods, i.e., the classic and elim, to identify the over-representation of GO terms (Alexa and Rahnenfuhrer 2018). The classic method analyzes GO identifiers in a standard way, in isolation from their hierarchical structure, therefore, it often reports more general GO terms as statistically significant. The elim method was designed to be more conservative then the classic method and therefore one expects the *p* values returned by the former method are lower bounded by the *p* values returned by the later method. Moreover, it tries to take into account the hierarchy of GO terms and focus on the most specific ones (Alexa et al. 2006; Alexa and Rahnenfuhrer 2018). In our analysis, we focused on statistically significant GO terms, indicated by *p* values below the standard significance level alpha = 0.05 and with more than 5 significant genes for both Fisher classic and Fisher elim methods (except for several GO terms with ≤ 5 significant genes, but substantially lower *p* values (*p* < 0.01), which are also shown in Table 3). Genes characterized by those GO terms demonstrated higher incidence in the analyzed gene set, than it would be expected at random.

## Results

### Sequencing

In order to investigate the genetic differences between virulent and avirulent *B. xylophilus* populations, four representative strains with different pathogenic traits were resequenced. After sequencing and filtering of low quality bases ca. 37 millions of read pairs for both the virulent populations (BxPt67OL and BxMad24C), and ca. 37 and ca. 35 millions of read pairs for the avirulent populations (C14-5 and OKD-1, respectively) have been obtained (Table 2). The obtained sequences were deposited at NCBI Sequence Read Archive (SRA) under accession number PRJNA630377.

**Table 2:** Summary of sequencing, clustering, and annotation results

Parameter	Virulent		Avirulent	
	BxPt67OL	BxMad24C	C14-5	OKD-1
Number of read pairs	37,299,205	37,227,358	37,160,862	35,259,088
Number of mapped reads	24,574,325	66,300,708	43,722,336	10,636,716
% of mapped reads	32.70	87.29	57.67	15.01
Coverage	36.0x	111.1x	66.7x	13.7x
Sequence variants (GATK)	1,161,206	1,049,303	2,156,336	1,926,667
High impact variants	5142	4102	8220	9090
Number of genes with high impact variants	2324	1838	3578	3831

After mapping to the *B. xylophilus* reference genome, for the virulent populations BxPt67OL and BxMad24C ca. 3.7 Gb and ca. 9.9 Gb paired end data were obtained, respectively. For the avirulent populations C14-5 and OKD-1 ca. 6.6 Gb and ca. 1.6 Gb paired end data were obtained, respectively. Overall, 32.70% and 87.29% reads of the virulent populations (BxPt67OL and BxMad24C, respectively) and 57.67% and 15.01% of the avirulent populations (C14-5 and OKD-1, respectively) were successfully aligned to the *B. xylophilus* genome. Variable levels of mapping of the examined populations to the *B. xylophilus* reference genome were due to the presence of random bacterial sequences in extracted DNA. Unmapped reads were analysed with the Kraken program (Wood and Salzberg 2014). The performed analysis showed that virulent populations BxPt67OL and BxMad24C contained 34.85% and 10.46% while the avirulent populations C14-5 and OKD-1 contained 27.93 and 56.53% reads assigned to the bacteria, respectively. Unfortunately, the share of bacterial data resulted in less available data derived from nematodes and, consequently, less coverage, especially for the population OKD-1.

### Sequence variants

The program GATK HaplotypeCaller has determined the sequence variants based on the paired-end reads mapped to the *B. xylophilus* reference genome. The genomes of *B. xylophilus* were found to be highly variable comparing to reference Ka4C1 strain; 1,161,206 and 1,049,303 variant positions for virulent BxPt67OL and BxMad24C, and 2,156,336 and 1,926,667 for avirulent C14-5 and OKD-1 populations were detected, respectively (Table 2). The results of the analysis showed that avirulent populations had 1.8 × more variants than virulent ones. The *B. xylophilus* reference genome corresponds to the virulent population, therefore the detection of fewer variants for the virulent populations seems to be as expected.

The ensembl variant effect predictor (VEP) program revealed the presence of variants with significant consequences for the protein function (i.e., high impact variants). Significant differences were found between virulent and avirulent populations. While the virulent BxPt67OL and BxMad24C populations had 5142 and 4102 high impact variants comparing to Ka4C1 strain, the avirulent C14-5 and OKD-1 had 8220 and 9090 high impact variants, respectively. Moreover, 2324 (BxPt67OL) and 1838 (BxMad24C) genes with high-impact variants were identified in virulent populations, and 3578 (C14-5) and 3831 (OKD-1) genes were identified in avirulent populations, respectively (Table 2; Table ESM1). Both analysed virulent populations differed from the reference strain by 469 genes with high-impact variants, while both avirulent populations differed by as many as 1576 genes (Table ESM1).

### GO-enrichment analysis

For the above genes, functional annotation was performed with topGO against Wormbase database to assign it to *B. xylophilus* molecular functions, biological processes and cellular components. Currently, 17,704 *B. xylophilus* genes are annotated in WormBase (WS277 release). In the presently reported research nematode gene annotations for 10,438 genes were determined (Table ESM2). For the avirulent populations, the GO identifiers distinguished 6 biological processes, 19 molecular functions and 1 cellular component for the avirulent populations, while for the virulent populations, the GO analysis distinguished 1 biological process, 3 molecular functions, and 1 cellular component (Table 3). For each GO category, genes associated with molecular functions, biological processes, and cellular components were also identified and presented in Tables ESM3, ESM4, ESM5, ESM6, ESM7 and ESM8.

**Table 3:** Enriched statistically significant GO terms (*p* < 0.05) for two avirulent and two virulent populations (the results are presented in ascending order according to Fisher's *p* value for the elim method)

Form	GO category	GO identifier	Term	Annotated	Significant	Expected	Rank in classic Fisher	elim Fisher	classic Fisher
Avirulent	Molecular function	GO:0004190	Aspartic-type endopeptidase activity	113	21	7.87	5	0.000028	0.000028
		GO:0005272	Sodium channel activity	54	12	3.76	8	0.00026	0.000026
		GO:0046923	ER retention sequence binding	3	3	0.21	9	0.00034	0.00034
		GO:0004222	Metalloendopeptidase activity	120	19	8.36	11	0.00058	0.00058
		GO:0016298	Lipase activity	31	7	2.16	13	0.00462	0.00462
		GO:0008271	Secondary active sulfate transmembrane transporter activity	17	5	1.18	14	0.00493	0.00493
		GO:0004198	Calcium-dependent cysteine-type endopeptidase activity	6	3	0.42	17	0.00574	0.00574
		GO:0070011	Peptidase activity, acting on l-amino acid peptides	522	73	36.37	2	0.00611	0.0000000029
		GO:0043492	ATPase activity, coupled to movement of substances	78	12	5.43	18	0.00728	0.00728
		GO:0016820	Hydrolase activity, acting on acid anhydrides, catalyzing transmembrane movement of substances	74	11	5.16	20	0.01281	0.01281
		GO:0042626	ATPase activity, coupled to transmembrane movement of substances	74	11	5.16	21	0.01281	0.01281
		GO:0015399	Primary active transmembrane transporter activity	75	11	5.23	22	0.01411	0.01411
		GO:0015405	P–P-bond-hydrolysis-driven transmembrane transporter activity	75	11	5.23	23	0.01411	0.01411
		GO:0004620	Phospholipase activity	30	6	2.09	26	0.01568	0.01568
		GO:0050660	Flavin adenine dinucleotide binding	57	9	3.97	27	0.01611	0.01611
		GO:0008233	Peptidase activity	546	78	38.04	1	0.01626	0.00000000027
		GO:0017171	Serine hydrolase activity	80	11	5.57	29	0.0221	0.0221
		GO:0008236	Serine-type peptidase activity	80	11	5.57	30	0.0221	0.0221
		GO:0004180	Carboxypeptidase activity	37	6	2.58	33	0.04077	0.04077
	Biological process	GO:0006508	Proteolysis	648	86	45.74	1	0.0000000013	0.0000000013
		GO:0006621	Protein retention in ER lumen	3	3	0.21	2	0.00035	0.00035
		GO:0035725	Sodium ion transmembrane transport	67	12	4.73	5	0.00221	0.00221
		GO:1902358	Sulfate transmembrane transport	16	5	1.13	7	0.00389	0.00389
		GO:0009435	NAD biosynthetic process	6	3	0.42	10	0.00594	0.00594
		GO:0071103	DNA conformation change	44	8	3.11	18	0.0107	0.0107
	Cellular component	GO:0016021	Integral component of membrane	4378	362	320.74	1	0.0000089	0.0000089
Virulent	Molecular function	GO:0016491	Oxidoreductase activity	483	19	9.58	1	0.003	0.003
		GO:0020037	Heme binding	120	7	2.38	2	0.0096	0.0096
		GO:0048037	Cofactor binding	153	7	3.03	10	0.032	0.032
	Biological process	GO:0055114	Oxidation–reduction process	521	19	10.76	1	0.0097	0.0097
	Cellular component	GO:0016020	Membrane	4608	114	98.91	1	0.0019	0.0019

*GO identifier (GO.ID)* identifier Gene Ontology, *Term* description of the GO identifier, *Annotated* the number of analyzed genes with a given GO identifier, *Significant* the number of genes belonging to the set genes with a given GO identifier, *Expected* the expected number of genes with a given GO identifier under condition that it is not statistically more frequent than in the set all genes with GO identifiers, *Rank in classic Fisher* Ranking of a given GO identifier by Fisher's *p* value for the classic method, *elim Fisher* Fisher's *p* value for the elim method, *classic Fisher* Fisher's *p* value for the classic method

For the biological processes, proteolysis, protein retention in ER lumen, sodium ion transmembrane transport, sulfate transmembrane transport, NAD biosynthetic process, and DNA conformation change were significantly enriched in the avirulent populations, whereas only oxidation–reduction process was enriched in the virulent populations.

For the avirulent populations, most highly represented terms of gene ontology (GO) in the molecular function category were related to endopeptidases (aspartic-type endopeptidase activity, metalloendopeptidase activity, calcium-dependent cysteine-type endopeptidase activity, peptidase activity and peptidase activity: acting on l-amino acid peptides) and other peptidases (serine-type peptidase activity, carboxypeptidase activity). The other molecular functions belonged to sodium channel activity, ER retention sequence binding, lipase activity, secondary active sulfate transmembrane transporter activity, ATPase activity: coupled to movement of substances and coupled to transmembrane movement of substances, hydrolase activity: acting on acid anhydrides and catalyzing transmembrane movement of substances, primary active transmembrane transporter activity, P–P-bond-hydrolysis-driven transmembrane transporter activity, phospholipase activity, flavin adenine dinucleotide binding, and serine hydrolase activity, whereas for the virulent populations oxidoreductase activity, heme binding, and cofactor binding were recorded.

For the cellular component, only integral component of membrane was significantly enriched in the avirulent populations, and in the virulent populations only membrane was found (Table 3).

## Discussion

The virulence mechanisms of *B. xylophilus* is complicated and involves many factors. It is suggested that *B. xylophilus* may use different genes or pathways to overcome the pine antinematodal response (Sommer and Streit 2011; Santos et al. 2012; Figueiredo et al. 2013). One of the approach to elucidate these mechanisms is whole-genome sequencing (Li et al. 2009).

In our study, four representative populations of *B. xylophilus*, two each of virulent and avirulent phenotypes, which originated from Japan and Portugal, respectively, were selected. All those populations presented different phenotypic or ecological traits and they had already been used in several previous studies (Aikawa et al. 2003; Filipiak 2015). The examined populations were closely related, and they shared some similar characteristics within the virulence category, including faster and slower development throughout the life cycle, in virulent (BxPt67OL and BxMad24C) and avirulent (C14-5 and OKD-1) populations, respectively.

Our genome-wide analysis revealed that virulent populations of *B. xylophilus* has high level of genome variation (BxPt67OL—1.56% and BxMad24C—1.41% comparing to reference genome). Differences were bigger when we compared avirulent populations to reference genome (C14-5—2.91% and OKD-1—2.60%). Obtained data are in line with genomic inter-strain differences reported by Palomares-Rius et al. (2015). It seemed that the level of diversity in the *B. xylophilus* genome was high but similar situation had been also observed in other hyper-diverse organisms, including nematodes (e.g., *Caenorhabditis brenneri* or *C. remanei*) (Cutter et al. 2013; Palomares-Rius et al. 2015; Ding et al. 2016). Previous studies indicated that organisms with large population size, short generation time, and small body size were more likely to be hyper-diverse (Cutter et al. 2013). *B. xylophilus* presents all the aforementioned characters. It is also possible to find some sequence polymorphisms among different *B. xylophilus* isolates originating from a local area (Ding et al. 2016).

Our genome analysis revealed also the presence of bacterial sequences in the sequencing data. It has previously been confirmed that *B. xylophilus* is associated with a range of bacterial species which might form an important component of the infection process (Vicente et al. 2012; Espada et al. 2016). A number of random bacteria is also frequently present in the nematode laboratory cultures. However, in this study we did not focus on this issue.

In the presently reported study, Gene Ontology (GO) was enriched in 6 terms involved in biological processes, 19 terms in molecular functions, and 1 term in cellular component for avirulent populations. In contrast, for virulent populations, Gene Ontology (GO) was enriched in 1 term involved in biological processes, 3 terms in molecular functions, and 1 term in cellular component. All these enriched terms were statistically significant (*p* < 0.05). The conducted study revealed that the examined avirulent populations contained more high impact variants and all GO categories (i.e., biological process, molecular function and cellular component) were significantly more enriched, especially those involved in molecular functions (Table 3).

Earlier research confirmed that the effective antioxidant ability is of critical importance in establishing the infection (Shinya et al. 2013; Vicente et al. 2015). Our study revealed that oxidoreductase activity (GO:0016491) and oxidation–reduction process (GO:0055114) were enriched in the virulent populations as statistically significant (Table 3). A high representation of these two terms may indicate a higher oxidative stress tolerance in virulent populations. In the early stages of invasion, *B. xylophilus* has to overcome host defense mechanisms, such as strong oxidative stress. It was previously confirmed that according to the functional annotation, some of the cell wall degradation-related genes were upregulated significantly. The oxidoreductase and hydrolase genes are considered to be key factors that allow *B. xylophilus* to invade its host (Qiu et al. 2013). Only successful, virulent nematodes are able to tolerate the plant defenses, and further migrate and proliferate inside of the host tree. However, it was also proved that different virulent *B. xylophilus* populations exhibited different tolerance to oxidative stress which is crucial component in host defense mechanisms (Vicente et al. 2015; Ding et al. 2016). Previous research suggested that catalases of highly virulent *B. xylophilus* were crucial for the nematode survival under prolonged exposure to oxidative stress in vitro (Vicente et al. 2015). Moreover, recent studies indicated that twelve antioxidant proteins were identified in the secretome of *B. xylophilus*. The secreted antioxidant enzymes would play an important role in *B. xylophilus* self-protection from oxygen free radicals in the pine tree (Kikuchi et al. 2011; Shinya et al. 2013; Ding et al. 2016).

To ensure successful infestation, *B. xylophilus* needs to break through pine host defense system. It is considered that reactive oxygen species (ROS) are to be the first line of defense in plants. Moreover, the suppression subtractive hybridization has revealed that the pathogenesis-related genes and cell wall-related genes induced by reactive oxygen species are crucial in the defense against PWN infestation (Nose and Shiraishi 2011; Hirao et al. 2012). Breaking down ROS defense also facilitate its ongoing and persistent infestations by weakening the resistance in host plants. ROS oxidize DNA, proteins, and lipids, which cause damage to organelles and inhibit cell functions in plant attackers. Among ROS, especially oxygen H_2_O_2_, is an important factor for regulation of host–nematode interactions and partly govern the success or failure of the disease (Li et al. 2016). Many reports suggest that nematode surface coat protein plays various crucial roles in host–parasite interactions, including regulation of microbes’ adhesion to the nematode’s body surface, lubrication, elicitation of the host defense responses, and modulation to help counter host defense responses (Spiegel and McClure 1995; Gravato-Nobre and Evans 1998; Shinya et al. 2010). *B. xylophilus* produces also surface coat proteins to help protect itself from ROS (Shinya et al. 2010, 2013). The identified surface coat proteins contained a potential regulator of ROS production, and ROS scavengers. These functions seem to be essential for establishing their infection and causing sudden death of host pine trees by the PWD (Shinya et al. 2010).

Another studies revealed that after inoculation of virulent populations of *B. xylophilus* into resistant Japanese black pine (*P. thunbergii*) more frequent accumulation of phenolic compounds around the cortex resin canals was observed. It was suggested that this accumulation was a very effective defense against infection due to restricting migration of *B. xylophilus* (Ishida et al. 1993). Hirao et al. (2012) assessed the difference in expressed sequence tag (EST) transcript diversity of activated defense genes and differences in the timing and magnitude of expression of these genes between resistant and susceptible *P. thunbergii* trees following PWN inoculation. In susceptible trees after PWN inoculation, pathogenesis related genes and antimicrobial-related genes were rapidly induced to high levels within 1-day post-inoculation. In contrast, a moderate defense response mediated by pathogenesis related protein expression followed by significant upregulation of cell wall-related genes induced by ROS was a very effective defense against PWN infection (Hirao et al. 2012). In similar research by Liu et al. (2017) studied the gene expression profiling between resistant and susceptible masson pines (*P. massoniana*) after inoculation with *B. xylophilus*. The resistant and susceptible phenotypes had a different defense mechanism in response to *B. xylophilus*. Detailed gene expression analysis suggested that terpenoids were prominent defense compounds against this nematode. Moreover, the higher activity of ROS-scavenging enzymes was effective to inhibit the death of resistant masson pines after PWN inoculation (Liu et al. 2017).

Earlier study revealed that genomic variants that introduce frameshift or stop codon mutations (i.e., high impact variants) could have serious effects on protein structures and functions (Palomares-Rius et al. 2015). High levels of possible loss of function may be related to proteolysis, which includes metalloendopeptidase activity, aspartic-type endopeptidase activity, and cysteine-type endopeptidase inhibitor activity (Palomares-Rius et al. 2015). In our research, metalloendopeptidase activity (GO:0004222) and aspartic-type endopeptidase activity (GO:0004190) were also over-represented for avirulent populations. It was confirmed that the GO term of aspartic-type endopeptidase activity enriched as a down-regulated function led to the low feeding activity (Palomares-Rius et al. 2015; Tanaka et al. 2019). Moreover, other, specific activity, such as hydrolase activity (GO:0016820) was also over-represented in possible loss-of-function variation. The fact that such variants are enriched in these genes may suggest that regions with such expansions are also subjected to changes in terms of point mutations or small insertions and deletions (Palomares-Rius et al. 2015).

It is known that peptidase families have a diverse range of biological roles, such as moulting, development, food digestion, and parasitism, in nematodes. Also in *B. xylophilus*, each gene in the expanded peptidase family has distinct role. This is a clear example of gene family evolution by gene duplication and functional divergence (Tanaka et al. 2019). Nematode peptidases, which hydrolyse polypeptides or proteins, participate in a wide range of molecular, biological, and cellular processes, such as a digestion of host proteins, moulting and embryonic development of the egg (Tanaka et al. 2019). The genome sequence of *B. xylophilus* revealed a large number of predicted peptidase genes (808 peptidase genes), representing the highest gene number among characterised nematode genomes. They include aspartic (106), metallo (230), cysteine (142), serine (170), threonine (13), unknown_32 (8), and unknown_69 (136) genes (Kikuchi et al. 2011; Tanaka et al. 2019). Moreover, according to Shinya et al. (2013) the GO analysis clearly showed the expansion of peptidases in the secretome of *B. xylophilus*. Especially, a large number of cysteine and aspartic peptidases were detected.

Based on these results and the previous study, one plausible explanation for the phenotypic differences between the virulent (high virulence and fast lifecycle) and avirulent (low virulence and slow lifecycle) populations is likely to be the lack of activities of effectors or digestive proteases. This could lead them to display low ingestion of nutrients and provoke a delay in development. Moreover, the effects of unique variations in specific genes could also be important in explaining the different ecological traits of avirulent populations (Palomares-Rius et al. 2015).

Our findings showed that the level of diversity in the *B. xylophilus* genome is high and comparable with that in other hyper-diverse organisms. We identified genes affected by genomic variation, and functional annotation of those genes indicated that some of them might have potential roles in pathogenesis. This comparative genome study with geographically distant *B. xylophilus* populations can facilitate understanding of the complex evolutionary/epidemic history of this pathogen. We believe that demonstrating genetic differences between virulent and avirulent populations will provide effective methods to distinguish these two nematode virulence forms at the molecular level. We hope that the presented data will facilitate a better understanding of molecular mechanisms of pine wilt disease and diagnose this nematode species. In turn, it may help to develop effective strategies for control of *B. xylophilus*. However, further research is needed to determine specific roles of these genes in the pathogenesis.

## Electronic supplementary material

Below is the link to the electronic supplementary material.

Detected genes with high impact variants for the studied populations (XLSX 200 kb)

Annotations for nematode genes (XLSX 336 kb)

Enriched GO terms for both avirulent populations—Molecular function (XLSX 1056 kb)

Enriched GO terms for both avirulent populations—Biological process (XLSX 1524 kb)

Enriched GO terms for both avirulent populations—Cellular component (XLSX 789 kb)

Enriched GO terms for both virulent populations—Molecular function (XLSX 1063 kb)

Enriched GO terms for both virulent populations—Biological process (XLSX 1187 kb)

Enriched GO terms for both virulent populations—Cellular component (XLSX 567 kb)

## Data Availability

Raw sequencing data presented in this paper are available under the NCBI Sequence Read Archive (SRA) accession number PRJNA630377.
